# Combating converging crises: The role of universities in global health, climate, and equity

**DOI:** 10.1016/j.joclim.2026.100683

**Published:** 2026-04-15

**Authors:** Jirair Ratevosian, Ama de-Graft Aikins, Zulfiqar Bhutta, Mercedes Bravo, Lara Dugas, Jessica Fanzo, Kimberly Fornace, Renzo Guinto, Diana Hernández, Allan Just, Thandi Kapwata, Lian Pin Koh, Katrina Korfmacher, Stephen Luby, Wuelton Monteiro, LaRon Nelson, Wendy O’Meara, William Pan, Robert Tighe, Chris Beyrer, Toddi Steelman

**Affiliations:** a Duke University, United States; b University of Ghana, Ghana; c University of Toronto, Canada; d University of Cape Town, South Africa; e School of Advanced International Studies (SIAS), The Johns Hopkins University, Bologna, Italy; f National University of Singapore, Singapore; g Brown University, United States; h South African Medical Research Council, South Africa; i University of Rochester, United States; j Stanford University, United States; k Amazonas State University, Brazil; l Yale University, United States; m The Aga khan University, South-Central Asia & East Africa

**Keywords:** Climate resilience, Health equity, Academic leadership, Transdisciplinary research, Global partnerships

## Abstract

An urgent, global challenge exists at the intersection of climate change, public health, and equity requiring sustained, interdisciplinary action. With political polarization, institutional disinvestment, uneven global commitments, misinformation and scientific denialism, and escalating climate-driven health risks, including heat-related illnesses, wildfires and floods, infectious diseases, mental health distress, and worsening health disparities, universities are uniquely positioned to drive innovative and transformative solutions. This perspective, based on an international convening held at the Rockefeller Foundation’s Bellagio Center in November 2024, outlines four interlinked roles for universities and offers a call to action. First, universities must sustain and protect scientific discovery, counter misinformation and safeguard research integrity. Second, they must diversify funding to maintain resilience. This means reimagining partnerships with governments, communities and industry. Third, they must uphold academic institutional responsibility including commitment to ethical inquiry, evidence-based science, and their own contributions to greenhouse gas emissions and sustainability. Fourth, they must transform education, equipping the next generation of leaders with transdisciplinary skills to navigate climate–health challenges. The recommendations provide a pathway during tenuous political times for academic institutions to remain trusted spaces for evidence-based solutions and global collaboration.

## Introduction

The impact of climate change on public health is widely recognized as an urgent global challenge [1,2]. By 2030, it is estimated that there will be US$2–4 billion a year in additional global direct health costs due to climate-related occurrences [3]. By 2050, projected global impacts on human health from climate change are estimated to cause an additional 14.5 million deaths on top of typical trends, contribute to the loss of more than two billion healthy life years, and $12.5 trillion in economic losses [4]. In addition, health care systems are expected to incur an additional $1.1 trillion in costs globally [4]. Increases in infectious diseases, respiratory and cardio-pulmonary disorders, undernutrition and malnutrition, heat-related illness and death, mental illness, more adverse birth and pregnancy outcomes, as well as injury, infection, and/or impact on livelihood resulting from extreme weather events are all anticipated as our planet warms [1,[5], [6], [7], [8]]. These impacts will disproportionately fall on the most vulnerable and under-resourced populations globally [4]. More than 3.5 billion people, nearly half the world’s population, are estimated to have high vulnerability to climate-related hazards [9]. In particular, Africa and southern Asia are more vulnerable than other regions to these climate-induced health impacts, given existing infrastructure, resource, population, and geographic inequities [4].

Addressing these climate-induced challenges is imperative. Human-caused warming is increasing at unprecedented rates according to existing records [10]. The Intergovernmental Panel on Climate Change (IPCC) originally estimated that warming would exceed 1.5C during the 21st century, which has already occurred, and projects additional increases from between 1.4–4.4C depending on the actions we take in the next years to decades [11]. 2024 was the hottest year on record going back to the industrial revolution [12]. Every additional fraction of warming amplifies the current extremes we are now facing. The rate of increase in carbon emissions slowed during 2014–2023 providing evidence that societal choices can make a difference, though this was partially due to the reduction in transportation and industry that occurred during the lock-down periods of the COVID-19 pandemic [10]. Reducing emissions is critical to preventing additional warming and associated catastrophic human health consequences into the future.

Climate change denial and disinformation remain a serious obstacle to global progress. In the United States (US), the January 20th, 2025 “Putting America First in International Environmental Agreements” Executive Order issued by President Trump put into motion the withdrawal of the United States from the Paris Agreement under the United Nations Framework Convention on Climate Change (UNFCCC) as well as the World Health Organization (WHO). The Trump Administration, through executive actions and agency rule-making, has escalated attacks on universities by threatening, pulling, freezing or delaying federal science funding, intimidating international scholars and students, and proposing restrictions on what universities can study and teach. These actions threaten not only university missions and operations, but also undermine global collaboration and advances in climate and health. Questions remain about the continued viability of international agreements and multilateral efforts at a time of growing nationalism, populism and retrenchments from global affairs [13]. These withdrawals and governance challenges underscore a broader political crisis, where growing geopolitical polarization and the erosion of trust in science threaten coordinated responses to global challenges [14]. Yet in the face of retreat and regression, bold, sustained collaboration across disciplines and borders is not just important—it’s the only path forward to confront the converging crises of climate and health. It has never been more important to assert the essential role of universities and the leadership they must exhibit.

Traditionally, universities have been well positioned to create and explore new scientific frontiers, push the boundaries of knowledge, and advocate for the adoption and implementation of established science and research. However, universities in the US now face new threats as significant funding cuts jeopardize critical research, limit interdisciplinary collaboration, and undermine their capacity to drive evidence-based climate and health solutions. These changes in the U.S. strongly impact global research and education as well.

This perspective asks a central question: *How can universities act as resilient scientific and civic actors capable of advancing climate–health equity in a period of political retreat and constrained resources?* To explore this question, the authors drew on a structured four-day convening held at the Rockefeller Foundation’s Bellagio Center in November 2024, shortly after the national U.S. elections. The meeting brought together 21 scholars, practitioners, and university leaders from across the Global North and South, representing expertise in climate science, public health, implementation research, ethics, sustainability, and university governance, at a moment of heightened political uncertainty and accelerating climate disruption. Through plenary sessions, small-group breakouts (2–3 people), and iterative synthesis discussions, participants debated key challenges, examined the evolving role of universities, and prioritized a set of strategic actions.

Priority areas were refined iteratively across sessions using thematic consolidation and a structured “scale of agreement” tool to assess convergence based on broad agreement rather than unanimity. Writing groups were then formed around prioritized themes to draft outlines, which were presented, critiqued, and revised in plenary. At the close of each day, facilitators and participants reviewed emerging themes, compared notes across groups, and identified areas of convergence and divergence. Each subsequent day began with a structured recap and validation of prior discussions, enabling iterative refinement and confirmation of alignment as the recommendations evolved. Thematic outputs from the convening were synthesized into the four action areas presented here: 1) sustaining and protecting scientific discovery, countering misinformation and safeguarding research integrity; 2) diversifying funding to maintain resilience; 3) upholding academic institutional responsibility including commitment to ethical inquiry, evidence-based science, and their own contributions to greenhouse gas (GHG) emissions and sustainability; and 4) transforming education to equip the next generation of leaders with transdisciplinary skills to navigate climate–health challenges.

## Roles and recommendations

Universities are mission-driven institutions deeply embedded in their local and global communities [15,16]. This stability ensures they can sustain long-term initiatives, often outlasting donor-reliant programs or government political and funding cycles. Roots within communities allow them to engage with local stakeholders, build trust, and tailor solutions to context-specific needs. Strong ties to communities are vital when addressing climate change and health disparities to ensure sustained, inclusive interventions that resonate with diverse populations.

### 2.1 Sustain and protect scientific discovery, counter misinformation and safeguard research integrity

The intersection of climate change and health presents an urgent global challenge necessitating the generation of actionable knowledge to address critical unknowns and advance responsive programming. Intellectual inquiry and academic freedom are distinct comparative advantages and cornerstones of universities [17,18]. Political interference in government research, profit-driven conformity among private entities, and harassment or intimidation aimed at suppressing unpopular views is common or increasing in some settings. Universities have been a distinct societal voice for protecting scientific integrity, providing protection from censorship, encouraging diverse perspectives, instilling creativity and innovation, and supporting critical thinking. Universities’ research resources, public service and community-driven missions, and independence set them apart from either the private sector or governmental research agencies in their ability to produce more comprehensive, equitable, inclusive, and accessible knowledge.

Climate denialism and misinformation continues to be pervasive in many parts of the world, eroding public and political support for urgent climate action [[19], [20], [21]]. This undermines investments in, as well as development and implementation of, science-driven policies that mitigate climate hazards on health, such as heat-related illnesses, vector-borne diseases, and mental health stressors exacerbated by extreme weather events [22,23]. Academic institutions provide credible information and counter misinformation through rigorous advances in basic science, implement findings in applied settings, interpret insights for broad audiences, and translate this knowledge into practice through university structures and partners [24]. As climate science is denied, manipulated, or otherwise undermined by nation-state actors, industry, and others with vested interests in maintaining the current fossil fuel-based operations of our societies, the university mission is relevant now more than ever.

Research on the impacts of climate change on human health is a rapidly evolving area of study, with some areas only in nascent stages of understanding. For example, researchers have been tracking the evolution of heat-related health impacts and the development of subsequent health service responses. A review in 2024 illustrated that heat-related impacts due to increasing temperatures have significantly worsened over time [25]. Extreme heat can cause an even larger range of issues including acute cardiovascular events, respiratory illnesses, and now even kidney disease with a two-year survival after diagnosis [25,26]. This kidney disease is rapidly emerging as a leading cause of death among outdoor laborers in South Asia and Central America [[27], [28], [29]]. This typifies how and where climate is disproportionately affecting poorer populations who rely on manual labor to support their families and who may not be empowered to effect responsive change in their communities [30]. Universities’ basic and applied research in these domains combined with their teaching and convening missions position them to amplify solutions to improve societal impact, especially where universities are part of health systems and medical centers.

Universities often have state-of-the-art research facilities, field sites, archives, libraries, computing resources, datasets, and other infrastructure that can be leveraged by a broad research community. The public service oriented mission of universities, that does not necessitate profit, means there is more leeway to consider often ignored and under-explored perspectives such as the disproportionate impacts on marginalized communities, equity and ethical considerations, community interventions, and the mental health effects associated with climate anxiety and trauma. Universities often also have more space to fail in experimentation and to learn, a critical tenet of academic inquiry.

Implementation science is another important function that universities directly or indirectly support with local partners to advance novel mitigation, long-term resilience, and health equity-promoting strategies. Research grounded in real-world settings that rapidly evaluates and advances community-level mitigation and adaptation climate-health interventions for programming is key to the global response [37]. Through working in partnership with local partners, stakeholders and rightsholders, respecting their partners’ contexts, and rapidly sharing information to meet the needs of locally defined problems and solutions, universities can accelerate the cycle of learning and innovation on the ground.

Finally, universities also have played important roles in traversing geopolitical divides by providing a soft power avenue as part of science diplomacy [38]. In an increasingly polarized and divided world, universities remain a center where a vibrant, connected global scientific community can flourish. Yet these contributions depend on protecting the very foundation of academic freedom and research funding. Defending open inquiry from political interference and funding instability is a precondition for all further action. At the same time, universities must recognize their own vulnerabilities—shrinking budgets, donor dependence, and the risk of being isolated from public trust—and develop diversified, resilient funding models.

### 2.2 Diversify funding to maintain resilience through partnerships with governments, communities and industry

As the funding model for universities changes, universities will need to be more creative, flexible and entrepreneurial in how they fund their research and activities. Partnerships and collaboration with other sectors will be essential. Universities are international institutions that attract a diversity of faculty, staff, and students with the potential for research and engagement that span across the globe [39]. However, given the current funding environment, universities will achieve optimal impact with stronger partnerships, consortia, and communities of practice with other universities, non-profit organizations, philanthropic organizations, and the private sector as part of a more collaborative, networked ecosystem.

Universities generate knowledge to inform and support climate-health policy at local, national, and international levels. Universities bridge the gaps between academic research and practical applications through engaging with policymakers, normative bodies, non-governmental organizations, the private sector, and other stakeholders. They do so as participants and as convenors of advisory groups, consultancies, and planning processes. Supported by university driven research, the U.S. National Academies of Sciences, Medicine, and Engineering, and the World Health Organization, among others, have developed research agendas to guide the direction and focus of global research that could address issues such as these extreme heat-driven morbidities [40,41]. These efforts will now need to attract private, non-profit or philanthropic support to complement or supplement funding that otherwise might have come from national sources. Private industry can also be an additional voice for lobbying governments to support these strategic policy efforts. University faculty and staff provide technical, scientific and policy expertise that complements the capacity in other sectors to provide advice and insight into vulnerability and adaptation assessments, national adaptation plans, global partnerships, such as the Global Environmental Facility and Green Climate Fund, and global multilateral agreements like UNFCCC, as well as the Alliance for Transformative Action on Climate and Health (ATACH) [32,42].

Inequities characterize funding availability for researchers in the Global North and Global South. Populations in the Global South are disproportionately affected by the changing climate; however, most funding sits in the Global North [43]. For example 70% of noncommunicable disease deaths occur in low and middle income countries (LMICs) but only 0.6% of research funding on noncommunicable diseases is focused in LMICs [44]. Consortia between the Global South and North can empower vulnerable and marginalized voices to lead on climate and health research agendas, which should be led by institutions situated in their own contexts [45,46]. While consistent funding remains challenging, partnerships with collaborating universities can provide meaningful capacity building within the Global South. South to South collaboration is also increasing through efforts across UN agencies, university consortia, and funders [[47], [48], [49], [50], [51]]. Funding programs that promote more equitable partnerships between mid-career researchers in sub-Saharan Africa and the United Kingdom, such as the AREF-MRF Equitable Partnership Programme [52], recognize North-South power dynamics and call attention to them.

Key areas for strategic intervention could catalyze funding interest from diverse sectors. In 2025, there is increased urgency for universities or other trusted sectors to steward data, by both creating and coordinating climate-health data repositories to ensure access to international datasets [[53], [54], [55]]. In the U.S., datasets and web-based tools have been removed from federal agency sites or disabled due to staff layoffs and terminations. Long-standing hubs for climate and health data and information, such as at the U.S. Department of State, Environmental Protection Agency, National Oceanic and Atmospheric Administration (NOAA), U.S. Agency for International Development and the National Institutes for Health (NIH), have stopped collecting and analyzing data needed to inform climate and health responses [56]. For instance, the NOAA climate-science hub (climate.gov) team was fired on May 31, 2025, the website relocated and will no longer post current content [57]. The website of the U.S. Global Change Research Program, which houses the National Climate Assessment reports, were taken offline on July 1, 2025 and federal staff who coordinate the report were fired [58]. The National Institutes of Health ended its Climate Change and Health Initiative and stopped funding research on the health effects of climate change [59,60]. In April 2025, the Centers for Disease Control and Prevention cut the entire permanent staff of the Division of Environmental Health Science and Practice, which supports several climate and health-relevant initiatives, including the Environmental Public Health Tracking program [61,62]. Joint university intervention along with data sharing protocols and agreements are one response to these rapidly evolving conditions to preserve data and access.

Further, universities can create, develop, and provide collaborative advantages to multi-country and multidisciplinary climate and health datasets. Developing standards and integrating climate and health data is vital for understanding basic mechanistic relationships and trends, crafting mitigation and adaptation interventions, and building multi-level and multi-scale resilience. Multi-country datasets with these characteristics help promote comparative analysis for both country specific and global patterns of understanding. However, challenges persist such as reconciling spatial and temporal mismatches between datasets, ensuring data accessibility for affected communities, connecting health and meteorological data and communication, and generating interoperable, comprehensive datasets tailored to regional needs [63,64], as well as ethical issues regarding the access to participant level data.

The climate–health crisis cannot be solved by universities alone. Academic institutions should actively collaborate with local and national governments to embed research into municipal climate resilience plans, health adaptation strategies, and early-warning systems. Partnerships with public health agencies and city governments can translate data into action, particularly for communities facing heat, flooding, and infectious disease threats. This is also an opportunity to reframe climate–health solutions as investments in building adaptive, thriving societies—language that resonates with policymakers across the political spectrum.

Universities also have convening power that can bring business, philanthropy, and civil society to the table. Industry partnerships can catalyze investment in climate-health data infrastructure, co-fund translational research, and advocate for enabling policies that scale solutions. Universities can serve as trusted intermediaries in this context, designing tiered access models and secure data environments that balance legitimate data protection concerns with the need for equitable, cross-border scientific collaboration. In a world of growing nationalism and increased data security concerns, establishing new networks with access to climate-related data will need to be attentive to data sensitivities, and specify ownership structures and access as well as compliance measures. By engaging civil society organizations and frontline communities, universities can ensure that solutions are equitable, culturally responsive, secure, and grounded in lived experience.

### 2.3 Advance institutional responsibility by leading on ethics, equity, community engagement, and sustainability

Institutional responsibility includes several areas: commitment to academic inquiry and evidence-based science; serving local communities and tackling inequities; addressing ethical issues related to research and services; and addressing universities’ own contributions to GHG emissions and sustainability. Universities are themselves communities in which people live, work, study, and play [65]. They can experiment with implementation of sustainable practices in their own environments, including energy use, transportation, landscape management, food services, purchasing, and waste management [65,66]. Universities can develop and test approaches that address climate resilience and health equity on a manageable scale, offering real-world insights that can be adapted and implemented elsewhere [[67], [68], [69]]. These activities can provide training grounds for the next generation of climate and health leaders.

Ideally, universities foster neutral, unbiased objective, transparent science delivery. The basic and applied science missions of universities prioritize intellectual freedom, long-term inquiry, and openness and sharing with the broader scientific community and the public—characteristics that lead to greater credibility and trust—especially when coupled with commitment to high-quality, peer-reviewed information sharing and knowledge transparency. Given academic independence, universities tend to have greater trust than other types of institutions, although this may be faltering in places like the U.S․ [70]. Climate and health research that leads to short- and longer-term positive impact is one pathway for universities to reinforce their credibility and trustworthiness.

Equity is at the core of all climate and health crises. Equitable health solutions include deep engagement with local communities as part of the health delivery mission. Research and teaching hospitals often serve patients, communities, and populations that bear the brunt of climate impacts, including for morbidities related to extreme heat, toxic exposures, expanding infectious diseases, and mental distress, among others. Linking patient services to research and training is the lifeblood of teaching hospitals, and this central role will likely only increase as the climate crisis worsens. Schools of medicine, nursing, health systems, public health, and other health disciplines, as well as other university-affiliated entities, are often embedded in underresourced communities. As anchor institutions in their communities, they may contribute to creating early warning systems, mapping and tracking climate driven diseases, developing transdisciplinary interventions, and engaging with affected residents [71].

Ethical considerations such as unintended consequences or biases of research, equity and fairness in interventions, and the moral obligation to address health burdens are becoming increasingly important in climate-health research [72]. Universities are well positioned to demonstrate best practices, where transparency, inclusivity, and advocacy for global data equity are part of the norm. Greater transparency of and demonstrated and visible commitment to ethical considerations can reassure a skeptical public and further enhance trust by helping ensure fairness, equity, and the minimization of harm [73,74]. The collapse of recent studies precipitated by the dismantling and immediate withdrawal of funding by USAID, for instance, has left research participants mid-way through treatment regimens, or worse still, outfitted with experimental devices [75]. Elimination of “Diversity, Equity, and Inclusion” research funding by federal agencies in the U.S. has threatened the future of health equity research, with a particular focus on curtailing environmental justice initiatives [76]. Universities can build capacity of local partners to take on the leadership roles in managing diversified, more sustainable funding models to avoid reliance on what is rapidly becoming less stable, larger donor resourcing. Emerging work in planetary health and One Health ethics urges institutions to move beyond narrow, human-centered frameworks and adopt ethical approaches that account for environmental integrity, intergenerational justice, and systemic inequities [77,78].

Lastly, academic health systems are also responsible for greenhouse gas emissions and therefore must take accountability for associated solutions [79]. Globally, the health care sector is responsible for almost 4.4% of carbon emissions [80,81]. Many universities and academic health care systems are working to reduce greenhouse gas emissions [67,82]. As of December 2022, 53% of academic health care systems in the US had taken steps to set specific net zero emission targets, tracking progress, and putting in place other mitigations such as telemedicine, reducing health care waste, creating more efficient clinical protocols, and decarbonizing energy use [83]. These efforts may innovate cost-effective approaches and serve as a model for other health systems. Universities in other countries are taking similar actions and could motivate others to join [84].

Beyond these discrete areas of responsibility, universities play a critical institutional role as stewards of scientific integrity, ethical practice, and public trust—roles that are increasingly under strain in polarized political and funding environments. Science is a messy practice and not easily communicated. Co-creation with key partners can help in the translational mission while also building credibility. Working hand in hand with health systems, public health agencies, emergency services and industry can create better opportunities to serve society well while also conveying the complexities associated with knowledge creation and discovery.

### 2.4 Transform education to equip the next generation with transdisciplinary skills for climate–health leadership

Universities play a pivotal role in educating the next generation of climate and health problem solvers and leaders [85]. Universities have made strides in developing transdisciplinary environmental studies and sustainability education but need to be strengthened and expanded to include a greater emphasis on health and equity. In 2020, only 15% of medical schools were teaching about the intersection of climate change and health [86], and while this effort has been growing, it has not been fast enough. By integrating climate and health topics into their curricula, universities equip students with the knowledge and skills necessary to address the components outlined in this article, including academic inquiry and innovation, multi-sectoral and interdisciplinary collaboration, service and support to communities, and institutional responsibility. This will require a new, concerted effort to develop the workforce, such as the multi-institutional collaborative online courses hosted by Columbia University [87]. Ideally, climate and health issues would be embedded in all core disciplines of learning to normalize attention to these issues.

University programs that equip faculty to empower and inspire students with the tools needed to tackle global climate, health, and equity challenges through collaborative, solutions-oriented training enable the next generation to rapidly develop and implement solutions [88]. Programmatic content would focus on key strategic, interdisciplinary areas such as (1) literacy in climate science, impacts, and policy; (2) applied knowledge of climate impacts on health, identifying vulnerable communities, and epidemiological science; (3) understanding the importance of equity in climate-health relationships, including how it can be measured and how it can be improved; (4) developing interdisciplinary skills; and (5) building capacity to develop, implement and evaluate interventions and contribute to climate solutions through communities, technology, policy, business or other pathways. This effort would also address the need for more equitable access to education and shared learning across countries and regions of the world through networks such as the Climate-Health Africa Network for Collaboration and Engagement (CHANCE) [89].

Universities are particularly well positioned to engage in interdisciplinary research efforts given the breadth of disciplines represented within singular institutions, as well as end-to-end research pipelines where work can be conducted from basic science through policy and implementation. For example, the interdisciplinary Heat Adaptation Benefits for Vulnerable groups in Africa (HABVIA) study in South Africa is a multi-country exploration leveraging four existing community-based cohorts to examine the efficacy of a housing adaptation (reflective roof paint) to improve non-communicable disease outcomes from heat stress [31]. Notably, studies such as HABVIA not only include climate science, but also basic and clinical health research as well as bring expertise in human behavior, social and political theory, and community participatory research. An increasing number of interdisciplinary institutes and funding mechanisms focused on climate and health exemplify similar potential for interdisciplinary learning [[32], [33], [34], [35], [36]].

Alongside curricular expansion and research training, universities carry a broader responsibility to shape the next generation of climate–health leaders capable of operating across science, policy, and practice. Embedding climate–health curricula across disciplines, offering joint degrees and experiential learning opportunities, and creating cross-sector fellowships will produce graduates equipped to navigate political, economic, and social complexities. Education that explicitly addresses equity, governance, and implementation challenges will prepare future leaders to sustain progress when political conditions shift.

## Conclusion

We are at a pivotal moment in the climate crisis. The year 2024 was the hottest on record, and humanity experienced a cascade of climate-related disasters including unrelenting heatwaves, catastrophic floods, mega-storms, and wildfires of unprecedented impact [90]. In 2025, the crisis was compounded by sharp reversals in U.S. climate leadership, rising populism and disinformation, ongoing challenges to global climate diplomacy - including finance gaps - and the further marginalization of the most vulnerable. At the same time, universities are operating under intensifying strain, marked by declining public trust, constrained and volatile funding, heightened political scrutiny, and internal competition across missions that limit institutional agility and risk tolerance. These pressures complicate universities’ ability to sustain long-term climate engagement, invest in interdisciplinary work, and translate knowledge into action at scale. The way forward must therefore confront these constraints directly—recognizing resource limitations, competing institutional priorities, and political pressures—and move beyond aspiration toward pragmatic, adaptive strategies and implementation pathways designed to operate within these realities (see Table 1).

**Table 1:** Climate, Health and Equity Action Steps for Universities.

Strategic Area	Mechanisms / Example Actions	Illustrative Partners
**Sustain and Protect Scientific Discovery**	• Safeguard academic freedom and research integrity • Develop diversified funding models (philanthropy, multilateral grants) • Build cross-institutional research consortia for climate–health science	National science foundations, philanthropic donors (e.g., Rockefeller Foundation), multilateral agencies (WHO, UN), peer universities
**Reimagine Partnerships with Governments, Private Sector& Communities**	• Embed research in local climate resilience and health adaptation plans • Co-develop early-warning systems and risk mapping • Provide decision-support tools to policymakers • Engage private sector in co-funding translational research • Convene multi-sector coalitions to advocate for enabling climate–health policy • Partner with civil society to ensure equity and accountability	Municipal governments, public health departments, ministries of environment/health, community-based organizations, corporations (energy, health, tech), and philanthropic alliances
**Uphold Academic Institutional Responsibility**	Recommit to ethical and open inquiry, engage fully as a partner in health delivery, reduce GHGs and enhance sustainability efforts	Health systems, public health agencies, emergency management service providers, climate tech
**Transform Education for the Next Generation**	• Integrate climate–health modules into core curricula across disciplines • Offer interdisciplinary joint degrees and experiential learning opportunities • Train students as implementation scientists and policy advocates	Professional schools (medicine, public health, engineering), student groups, education networks, international education consortia

**Fig. 1:**
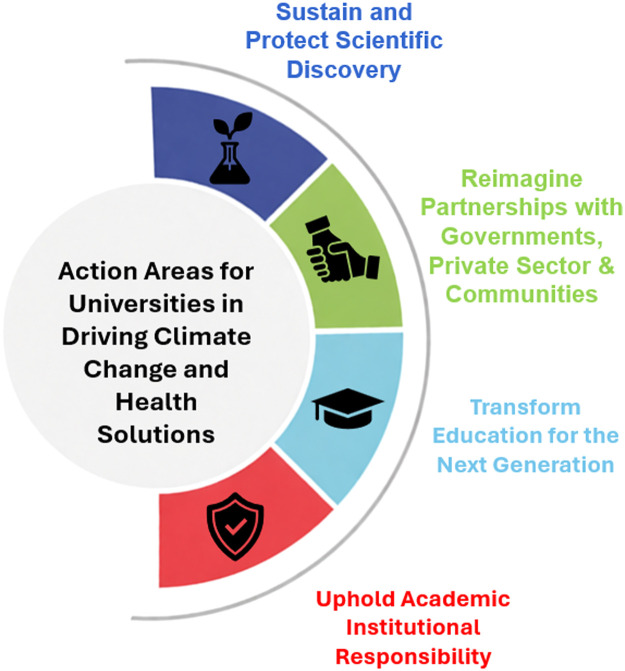
Action Areas for Universities in Driving Climate Change and Health Solutions.

Here, we present four recommendations for universities to confront the converging crises of climate and health (see Fig. 1). These efforts center equity, ensuring that those most affected by climate change and health disparities are not left behind. Climate-driven health inequities deepen existing social and economic divides. By fostering meaningful partnerships and amplifying underrepresented voices, universities can challenge power asymmetries and shape policies that advance climate and health justice.

In an era of political polarization and shrinking trust in science, universities remain one of the few enduring institutions capable of convening diverse actors around evidence and solutions. Acting on these priorities will require universities to defend academic freedom, diversify and stabilize funding, and embed their work within broader ecosystems that include governments, industry, civil society, and affected communities. The recommendations presented here are intentionally adaptive — recognizing that strategies must evolve alongside shifting political contexts, resource constraints, and climate impacts. By committing to this agenda, universities can reaffirm their role as trusted civic and scientific actors and help lead the world toward a more just, climate-resilient future.

## CRediT authorship contribution statement

**Jirair Ratevosian:** Writing – original draft, Supervision, Project administration, Conceptualization. **Ama de-Graft Aikins:** Writing – review & editing. **Zulfiqar Bhutta:** Writing – review & editing, Methodology. **Mercedes Bravo:** Writing – review & editing. **Lara Dugas:** Writing – review & editing. **Jessica Fanzo:** Writing – review & editing. **Kimberly Fornace:** Writing – review & editing. **Renzo Guinto:** Writing – review & editing, Writing – original draft, Conceptualization. **Diana Hernández:** Writing – review & editing. **Allan Just:** Writing – review & editing. **Thandi Kapwata:** Writing – review & editing. **Lian Pin Koh:** Writing – review & editing. **Katrina Korfmacher:** Writing – review & editing, Methodology. **Stephen Luby:** Writing – review & editing. **Wuelton Monteiro:** Writing – review & editing. **LaRon Nelson:** Writing – review & editing. **Wendy O’Meara:** Writing – review & editing. **William Pan:** Writing – review & editing. **Robert Tighe:** Writing – review & editing. **Chris Beyrer:** Writing – review & editing, Writing – original draft, Supervision, Funding acquisition, Conceptualization. **Toddi Steelman:** Writing – review & editing, Writing – original draft, Supervision, Funding acquisition, Conceptualization.

## Declaration of competing interest

The authors declare the following financial interests/personal relationships which may be considered as potential competing interests:

Financial support and travel were provided by The Rockefeller Foundation.
